# Poly-γ-Glutamic Acids Contribute to Biofilm Formation and Plant Root Colonization in Selected Environmental Isolates of *Bacillus subtilis*

**DOI:** 10.3389/fmicb.2016.01811

**Published:** 2016-11-10

**Authors:** Yiyang Yu, Fang Yan, Yun Chen, Christopher Jin, Jian-Hua Guo, Yunrong Chai

**Affiliations:** ^1^Department of Plant Pathology, Nanjing Agricultural UniversityNanjing, China; ^2^Department of Biology, Northeastern UniversityBoston, MA, USA; ^3^Institute of Biotechnology, Zhejiang UniversityHangzhou, China; ^4^Engineering Center of Bioresource Pesticide in Jiangsu Province, Key Laboratory of Integrated Management of Crop Diseases and PestsNanjing, China

**Keywords:** *Bacillus subtilis*, poly-γ-glutamic acid, biofilm formation, root colonization

## Abstract

*Bacillus subtilis* is long known to produce poly-γ-glutamic acids (γ-PGA) as one of the major secreted polymeric substances. In *B. subtilis*, the regulation of γ-PGA production and its physiological role are still unclear. *B. subtilis* is also capable of forming structurally complex multicellular communities, or biofilms, in which an extracellular matrix consisting of secreted proteins and polysaccharides holds individual cells together. Biofilms were shown to facilitate *B. subtilis*–plant interactions. In this study, we show that different environmental isolates of *B. subtilis*, all capable of forming biofilms, vary significantly in γ-PGA production. This is possibly due to differential regulation of γ-PGA biosynthesis genes. In many of those environmental isolates, γ-PGA seems to contribute to robustness and complex morphology of the colony biofilms, suggesting a role of γ-PGA in biofilm formation. Our evidence further shows that in selected *B. subtilis* strains, γ-PGA also plays a role in root colonization by the bacteria, pinpointing a possible function of γ-PGA in *B. subtilis*–plant interactions. Finally, we found that several pathways co-regulate both γ-PGA biosynthesis genes and genes for the biofilm matrix in *B. subtilis*, but in an opposing fashion. We discussed potential biological significance of that.

## Introduction

The soil bacterium *Bacillus subtilis* is well-known for its ability to form structurally complex multicellular communities, known as biofilms (Branda et al., 2001; Hamon and Lazazzera, 2001; Kolter and Greenberg, 2006; Aguilar et al., 2007; Vlamakis et al., 2013). A number of environmental strains of *B. subtilis* are able to form biofilms on solid agar media and floating pellicles at the air-liquid interface (Branda et al., 2001; Hamon and Lazazzera, 2001). Both types of biofilms demonstrate sophisticated surface architectures. In *B. subtilis*, the surface structural complexity is often used as a qualitative measurement of biofilm robustness (Branda et al., 2001). *B. subtilis* also forms root-associated biofilms, which protect plants from infections by pathogenic bacterial species and fungi through multiple mechanisms (Bais et al., 2004; Chen et al., 2012, 2013; Beauregard et al., 2013); *B. subtilis* mutants deficient or impaired in biofilm formation were shown much less effective in plant protection (Morris and Monier, 2003; Bais et al., 2004; Chen et al., 2013). In the field of agriculture, a number of *B. subtilis* strains have been engineered and widely used as the so-called biological control agent (BCA) for plant protection (Emmert and Handelsman, 1999; Aliye et al., 2008; Ji et al., 2008).

**FIGURE 1:**
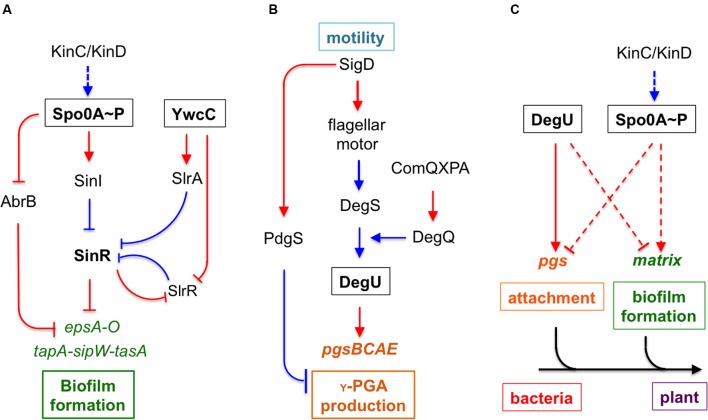
**An integrated model for regulation and function of poly-γ-glutamic acid (γ-PGA) and biofilm formation during *Bacillus subtilis*–plant interactions. (A)** A schematic drawing of the regulatory pathways in *B. subtilis* that control biofilm matrix genes. KinC and KinD are two sensory histidine kinases involved in sensing environmental signals. KinC and KinD activate the master regulator Spo0A through protein phosphorylation. Phosphorylated Spo0A in turn activates biofilm matrix genes via two independent mechanisms, one through the transition stage regulator AbrB and the other through the SinI-SinR regulatory module. SinR is a biofilm master repressor of the matrix genes. YwcC is a TetR-type repressor, which controls genes encoding two antagonist proteins (SlrA and SlrR) for SinR. The *epsA-O* and the *tapA-sipW-tasA* operons are involved in making the EPS and the TasA amyloid fibers, respectively, of the biofilm matrix. **(B)** The *pgsBCAE* operon for γ-PGA biosynthesis is chiefly regulated by the two-component system DegS-DegU. Another two-component system ComA-ComP regulates the *degQ* gene, which encodes a small regulator for DegU activation. DegU is also activated by a yet unknown mechanism in sensing flagellar motion. **(C)** The interplay between the control on biofilm formation and that of γ-PGA production. Response regulators DegU and Spo0A both mediate opposing regulation on γ-PGA biosynthesis genes and genes for biofilm matrix production in two independent pathways. Dashed lines indicate indirect regulations. We also propose that γ-PGA and biofilm matrix may play sequential roles in the attachment of the bacteria to the root surface and in formation of root-surface associated biofilms during *B. subtilis*–plant interactions. Lines in red indicate gene regulation while lines in blue indicate protein–protein interactions.

Bacterial biofilm formation depends on production of secreted polymeric substances, known as extracellular matrix (Branda et al., 2005). In *B. subtilis*, the matrix consists primarily of an exopolysaccharide (EPS), protein fibers (TasA and TapA), and a hydrophobin coat (BslA) (Kearns et al., 2005; Branda et al., 2006; Romero et al., 2010, 2011; Kobayashi and Iwano, 2012; Hobley et al., 2013). The regulatory pathway for biofilm matrix production is well-studied in *B. subtilis* (**Figure 1A**; Vlamakis et al., 2013). Multiple sensory histidine kinases (e.g., KinC/KinD) sense environmental signals, including those from the plants (López et al., 2009; McLoon et al., 2011; Chen et al., 2012; Beauregard et al., 2013; Shemesh and Chai, 2013). The kinases in turn activate the global master regulator Spo0A by protein phosphorylation via a phosphorelay (Burbulys et al., 1991; Jiang et al., 2000). Phosphorylated Spo0A (Spo0A∼P) induces biofilm formation via at least two independent mechanisms, in which the two master repressors SinR and AbrB that directly repress biofilm matrix genes are antagonized (Hamon and Lazazzera, 2001; Chai et al., 2011). In previous studies, a TetR-type transcription repressor YwcC was also shown to be involved in biofilm formation in *B. subtilis* (**Figure 1A**; Kobayashi, 2008; Chai et al., 2009).

*Bacillus subtilis* is also known to produce other secreted polymeric substances, including poly-γ-glutamic acids (γ-PGA), a polymer with the size ranging from ∼10 to ∼1000 kDa (Ashiuchi et al., 1999; Ashiuchi and Misono, 2002; Morikawa et al., 2006; Poo et al., 2010; Ogunleye et al., 2015). Because this polymer is biodegradable, γ-PGA may have important applications in the medical field and industry (Ashiuchi and Misono, 2002; Poo et al., 2010; Ogunleye et al., 2015). The γ-PGA biosynthesis genes are highly conserved in various *Bacillus* species. Several related *Bacillus* species have been shown to produce this polymer as well (Gardner and Troy, 1979; Makino et al., 1989; Stanley and Lazazzera, 2005). For instance, *Bacillus anthracis* produces γ-PGA as a surface-anchored polysaccharide and a part of the bacterial capsule (Makino et al., 1989). γ-PGA is also important for bacterial virulence in *B. anthracis* (Jang et al., 2011). In *B. subtilis*, biosynthesis of γ-PGA relies on the conserved operon *pgsB-pgsC-pgsA-pgsE* (originally named as *ywsC-ywtA-ywtB-ywtC*; hereafter the *pgs* operon) (Ashiuchi and Misono, 2002). A fifth gene *pdgS* (also as *ywtD*) next to the operon was recently characterized to encode a depolymerase for γ-PGA degradation (Suzuki and Tahara, 2003). Mutations in the *pdgS* gene result in a higher yield of γ-PGA and larger sizes of the produced polymers (Suzuki and Tahara, 2003). In *B. subtilis*, the *pgs* operon is regulated by two sets of two-component systems in cascade, DegS-DegU and ComA-ComP (**Figure 1B**; Stanley and Lazazzera, 2005; Chan et al., 2014). ComA-ComP is part of the quorum-sensing mechanism in *B. subtilis* involved in competence development (Hamoen et al., 2003). Upon activation by a peptide-based quorum-sensing signal (ComX), the response regulator ComP activates *degQ*, which encodes a small protein facilitator important for the phosphor-transfer from the histidine kinase DegS to the response regulator DegU (Msadek et al., 1991). DegU directly activates the *pgs* operon (Ohsawa et al., 2009). Interestingly, two recent studies independently showed that activities of DegU were elevated significantly in the mutant of *motB*, a gene encoding the flagella stator protein (Cairns et al., 2013; Chan et al., 2014). This implies that by a yet-unknown mechanism, the DegS kinase senses a signal linked to flagella motion when activating DegU. Expression of the *pgs* operon is also shown to be dependent on a small regulatory protein SwrA, originally identified as a regulator for swarming motility in *B. subtilis* (Kearns et al., 2004; Stanley and Lazazzera, 2005). It is not entirely clear how SwrA positively regulates the *pgs* operon. One study suggested that SwrA enhances DegU-mediated transcriptional activation of the *pgs* operon in *B. subtilis* (Osera et al., 2009).

In *B. subtilis*, although γ-PGA is a secreted polymeric substance, similar to the biofilm matrix components (e.g., EPSs), whether γ-PGA becomes part of the biofilm matrix and plays a structural role in biofilm matrix assembly is unclear. One previous study showed that the deletion mutation in the γ-PGA biosynthesis genes had no influence on the biofilm phenotype in the *B. subtilis* model strain NCIB3610 (Branda et al., 2006). Other studies performed under different media conditions and with different biofilm settings suggested that γ-PGA influenced biofilm robustness (Stanley and Lazazzera, 2005; Morikawa et al., 2006). In this study, we presented new evidence to address this discrepancy. We demonstrated that in many environmental isolates of *B. subtilis*, γ-PGA plays a notable role in increasing the robustness and complex morphology of the colony biofilms. We showed that several genetic pathways co-regulate both γ-PGA biosynthesis genes and genes for production of the biofilm matrix in *B. subtilis*, but in an opposing fashion. Finally, we also investigated the possible role of γ-PGA in bacteria–plant interactions.

## Materials and Methods

### Strains, Reagents, and Media Conditions

*Bacillus subtilis* environmental isolates and *B. subtilis* NCIB3610 (hereafter referred as 3610) were routinely grown in Luria-Bertani (LB) broth. For colony biofilm development, LBGM (Shemesh and Chai, 2013) and MSgg (Branda et al., 2001) were used. All strains used in this study are listed in **Table 1**. When necessary, antibiotics were used at the following concentrations: 5 μg ml^-1^ chloramphenicol, 0.5 μg ml^-1^ erythromycin, 10 μg ml^-1^ kanamycin, 12.5 μg ml^-1^ lincomycin, 50 μg ml^-1^ spectinomycin, and 5 μg ml^-1^ tetracycline for *B. subtilis* strains. Chemicals were purchased from Sigma. Restriction enzymes were purchased from New England Biolabs (NEB). Oligonucleotides were purchased from Integrated DNA Technologies (IDT). DNA sequencing was performed at Genewiz (NJ, USA).

**Table 1:** Bacterial strains used in this study.

Strain	Genotype	Reference
PY79	A laboratory strain used as a host for transformation	Schroeder and Simmons, 2013
NCIB3610	An undomesticated wild strain capable of forming robust biofilms	Branda et al., 2001
SM21	Environmental strain isolated from rhizosphere soil in China	Wang et al., 2012
CYBS-9	Environmental strain isolated from rhizosphere soil in China	Chen et al., 2013
CYBS-11	Environmental strain isolated from rhizosphere soil in China	Chen et al., 2013
CYBS-12	Environmental strain isolated from rhizosphere soil in China	Chen et al., 2013
CYBS-13	Environmental strain isolated from rhizosphere soil in China	Chen et al., 2013
CYBS-14	Environmental strain isolated from rhizosphere soil in China	Chen et al., 2013
CYBS-21	Environmental strain isolated from rhizosphere soil in China	Chen et al., 2013
CYBS-22	Environmental strain isolated from rhizosphere soil in China	Chen et al., 2013
CYBS-23	Environmental strain isolated from rhizosphere soil in China	Chen et al., 2013
CYBS-25	Environmental strain isolated from rhizosphere soil in China	Chen et al., 2013
CYBS-26	Environmental strain isolated from rhizosphere soil in China	Chen et al., 2013
CYBS-27	Environmental strain isolated from rhizosphere soil in China	Chen et al., 2013
CYBS-54	Environmental strain isolated from rhizosphere soil in China	Chen et al., 2013
CY49	*amyE*::P*_hyspank_*-*mKate2* in 3610, Cm^R^	Chen et al., 2012
CY50	*amyE*::P*_hyspank_*-*mKate2* in CYBS54, Cm^R^	Chen et al., 2013
CY150	*amyE*::P*_hyspank_*-*mKate2* and △*pgsBCAE* in 3610, Cm^R^, Sp^R^	This study
CY151	*amyE*::P*_hyspank_*-*mKate2* and △*pgsBCAE* in CYBS54, Cm^R^, Sp^R^	This study
CY258	△*motA* in 3610, Mls^R^	This study
CY480	*amyE*::P*_pgsB_-lacZ::spc*, △*kinA*::*mls*, and △*kinB::km* in 3610	This study
CY481	*amyE*::P*_pgsB_-lacZ::spc*, △*kinC*::*mls*, and △*kinD::tet* in 3610	This study
CY482	*amyE*::P*_pgsB_-lacZ*::*spc*, △*abrB::km*, and △*epsH::tet* in 3610	This study
CY485	*amyE*::P*_pgsB_-lacZ::spc* and △*spo0A::km* in 3610	This study
CY486	*amyE*::P*_pgsB_-lacZ*::*spc* and △*degSU::tet* in 3610	This study
CY487	*amyE*::P*_pgsB_-lacZ*::*spc* and △*slrR::tet* in 3610	This study
CY488	*amyE*::P*_pgsB_-lacZ*::*spc*, △*ywcC::km*, and △*epsH::tet* in 3610	This study
FY5	△*pgsBCAE* in CYBS54, Spc^R^	This study
FY6	△*pgsBCAE* in 3610, Spc^R^	This study
FY8	△*pgsBCAE* in CYBS-9, Mls^R^	This study
FY11	△*pgsBCAE* in CYBS-13, Spc^R^	This study
FY12	△*pgsBCAE* in CYBS-14, Mls^R^	This study
FY14	△*pgsBCAE* in CYBS-27, Spc^R^	This study
FY16	△*pgsBCAE* in CYBS-25, Spc^R^	This study
FY17	△*pgsBCAE* in CYBS-26, Spc^R^	This study
FY19	△*pgsBCAE* in CYBS-21, Mls^R^	This study
FY20	△*pgsBCAE* in CYBS-23, Mls^R^	This study
FY27	△*pgsBCAE* in CYBS-22, Mls^R^	This study
FY313	*amyE*::P*_pgsB_-lacZ* in CYBS54, Spc^R^	This study
FY316	*amyE*::P*_pgsB_-lacZ* in SM21, Spc^R^	This study
FY317	*amyE*::P*_pgsB_-lacZ* in CYBS9, Spc^R^	This study
FY318	△*pgsBCAE* in CYBS11, Spc^R^	This study
FY319	*amyE*::P*_pgsB_-lacZ* in CYBS11, Spc^R^	This study
FY320	△*pgsBCAE* in CYBS12, Spc^R^	This study
FY321	*amyE*::P*_pgsB_-lacZ* in CYBS12, Spc^R^	This study
FY323	*amyE*::P*_pgsB_-lacZ* in CYBS13, Spc^R^	This study
FY324	*amyE*::P*_pgsB_-lacZ* in CYBS14, Spc^R^	This study
FY325	*amyE*::P*_pgsB_-lacZ* in CYBS21, Spc^R^	This study
FY327	*amyE*::P*_pgsB_-lacZ* in CYBS23, Spc^R^	This study
FY329	*amyE*::P*_pgsB_-lacZ* in CYBS22, Spc^R^	This study
FY330	*amyE*::P*_pgsB_-lacZ* in CYBS25, Spc^R^	This study
FY331	*amyE*::P*_pgsB_-lacZ* in CYBS26, Spc^R^	This study
FY333	*amyE*::P*_pgsB_-lacZ* in CYBS27, Spc^R^	This study
FC100	*amyE*::P*_hyspank_-slrR::spc* and △*slrR::tet* in 3610	Chai et al., 2010
RL3852	△*epsH::tet* in 3610	Kearns et al., 2005
RL4573	△*kinA::mls*, △*kinB::km* in 3610	McLoon et al., 2011
RL4262	△*kinC::mls* in 3610	McLoon et al., 2011
RL4569	△*kinD::mls* in 3610	McLoon et al., 2011
RL4637	△*degSU::tet* in 3610	Losick lab
RL5273	△*kinC::mls*, △*kinD::tet* in 3610	McLoon et al., 2011
SB505YC110	△*tasA::tet* in 3610*amyE*::P*_eps_-lacZ* in 3610, Cm^R^	Branda et al., 2006; Chai et al., 2008
YC295	△*ywcC::km* in 3610	Chai et al., 2009
YC299	△*ywcC::km*, △*epsH::tet* in 3610	Chai et al., 2009
YC668	△*abrB::km*	This study
YC1275	*amyE*::P*_pgsB_-lacZ* in 3610, Spc^R^	This study
YY54	△*pgdS::tet* in 3610	This study
YY170	△*pgsBCAE* in SM21, Spc^R^	This study
YY198	△*ywcC::km*, △*epsH::tet*, △*pgsBCAE::spec* in 3610	This study
YY199	△*ywcC::km*, △*epsH::tet*, △*slrR::mls* in 3610	This study
YY200	△*ywcC::km*, △*epsH::tet*, △*slrR::mls*, △*pgsBCAE::spec* in 3610	This study
YY208	△*spo0A::spec*, △*abrB::km* in 3610	This study
YY214	△*spo0A::spec* in 3610	This study
YY233	△*spo0A::spec*, △*epsH::tet*, △*pgsBCAE::mls* in 3610	This study
YY245	*amyE*::P*_SM21pgsB_-lacZ* in SM21, Spc^R^	This study

### Strain Construction

All insertion deletion mutations were generated by long-flanking PCR mutagenesis (Wach, 1996). The mutation was first constructed in the *B. subtilis* laboratory strain PY79 (Schroeder and Simmons, 2013) and then introduced into various environmental isolates of *B. subtilis* by genetic transformation following a protocol described previously (Gryczan et al., 1978). Primers used to generate deletion mutations in the *pdgS* gene and the *pgs* operon are described in Supplementary Table S1. To construct strains with the P*_pgsB_*-*lacZ* fusion integrated into the chromosome at the *amyE* locus, genomic DNA containing P*_pgsB_*-*lacZ* was prepared from YC1275 that was constructed in a previous study (Gao et al., 2015) and was introduced into different strain backgrounds by transformation. A similar promoter fusion (P*_SM21pgsB_*-*lacZ*) was created by using the primers P*_pgsB_*-F1 and P*_pgsB_*-R1 (Supplementary Table S1) and the genomic DNA prepared from the *B. subtilis* strain SM21 due to extreme similarity in the DNA sequences of the *pgs* promoter region from SM21 and 3610 (Supplementary Figure S1). PCR amplified promoter sequences were then cloned into the vector pDG1730 to make a promoter-*lacZ* fusion. The integration of the reporter fusion to the *amyE* locus of the strain SM21 and verification of such integration followed a protocol described previously (Gao et al., 2015)

### Colony Mucoidy and Biofilm Formation

For comparison of colony mucoidy, *B. subtilis* strains were streaked out on LB agar plates (+1.5% agar). The LB agar plates were freshly poured, air-dried in the Lamina Flow hood for 40 min, and sealed and kept at 4°C prior to use. After streaking inoculation, the LB plates were incubated at 37°C for 12 h. Images of the colonies were taken using a Leica MSV269 stereoscope. For comparison of colony biofilms, *B. subtilis* cells were first grown to exponential growth phase in LB broth and 2 μl of the culture was spotted to the biofilm inducing media LBGM or MSgg. The plates were incubated at 30°C for 2–3 days. Images of the colony biofilms were recorded similarly.

### γ-PGA Isolation

The method for γ-PGA isolation was modified from a previously published paper (Stanley and Lazazzera, 2005). Briefly, cells were grown in LB at 37°C for 24 h. The supernatant was collected, brought to pH 2.0 with concentrated sulfuric acid, and then incubated at 4°C overnight. Ethanol was added to the supernatant to a final concentration of 80% and the sample was incubated at -20°C for 10 min for γ-PGA precipitation. Samples were centrifuged at 4°C and 5000 rpm for 30 min. The resulting pellet containing γ-PGA was suspended in dH_2_O prior to analysis by gel electrophoresis. The γ-PGA samples were size-fractionated on 8% acrylamide gels. The gels were stained with 0.05% methylene blue in 3% acetic acid for 30 min, and de-stained sequentially in dH_2_O.

### Assays of β-Galactosidase Activities

Assays were conducted as previously described (Chai et al., 2009). Cells were cultured in LB or LBGM medium at 37°C in a water bath with shaking. One milliliter of culture was collected at each indicated time point and cells were centrifuged down at 5000 rpm for 10 min. Cell pellets were suspended in 1 ml Z buffer (40 mM NaH_2_PO_4_, 60 mM Na_2_HPO_4_, 1 mM MgSO_4_, 10 mM KCl, and 38 mM β-mercaptoethanol) supplemented with 200 μg ml^-1^ lysozyme. Resuspensions were incubated at 37°C for 15 min. Reactions were started by adding 200 μl of 4 mg ml^-1^ ONPG (2-nitrophenyl-β-D-galactopyranoside) and stopped by adding 500 μl of 1 M Na_2_CO_3_. Samples were briefly centrifuged down at 5000 rpm for 1 min. The soluble fractions were transferred to cuvettes (VWR), and absorbance of the samples at 420 nm was recorded using a Bio-Rad Spectrophotometer. The β-galactosidase specific activity was calculated according to the equation (Abs_420_/time × OD_600_) × dilution factor × 1000. Assays were conducted at least in triplicate.

### Root Colonization Assay

Cell colonization in the tomato rhizosphere by a 3610 derivative (CY49), a CYBS54 derivative (CY50) and the corresponding Δ*pgs* mutants (CY150 and CY151) was monitored. The tomato seeds were surface-sterilized with a 30 s treatment in 70% ethanol, followed by 15 min in sodium hypochlorite (10% active chlorine), and by three subsequent washing steps with sterile water for at least 15 min each. The seeds were pre-germinated on sterile moist filter paper and were then incubated in a growth chamber (16:8 h light/dark photoperiod). Tomato plantlets were transplanted 2 weeks later into pots filled with sterilized soil (by autoclave). The pots were arranged in a randomized block design in a greenhouse. The temperature was maintained at 25°C and the photoperiod was 16:8 h light/dark. *B. subtilis* strains were grown in LB broth at 28°C for 24 h in a shaker at 200 rpm. Cell suspensions were adjusted to a cell density of 5 × 10^7^ cells per ml. Twenty milliliters of the *B. subtilis* suspension was applied as irrigation to each pot. Rhizosphere samples were collected at 1, 3, 5, 7, 13, 21, and 30 days after inoculation. Four rhizosphere samples were analyzed per treatment at each sampling time. Each rhizosphere sample consisted of the total root system with tightly adhering soil of four individual plants, which were thoroughly mixed and immediately processed for C.F.U. counts. To determine the C.F.U. counts of the inoculants by the plating method, serial dilutions of the cell suspension were spread-plated on LB agar plates with appropriate antibiotics and 100 μg ml^-1^ cycloheximide. Plates were incubated at 30°C overnight. The number of individual colonies was counted and the collected data were analyzed.

## Results

### Different Environmental Isolates of *B. subtilis* Vary Significantly in γ-PGA Production

**FIGURE 2:**
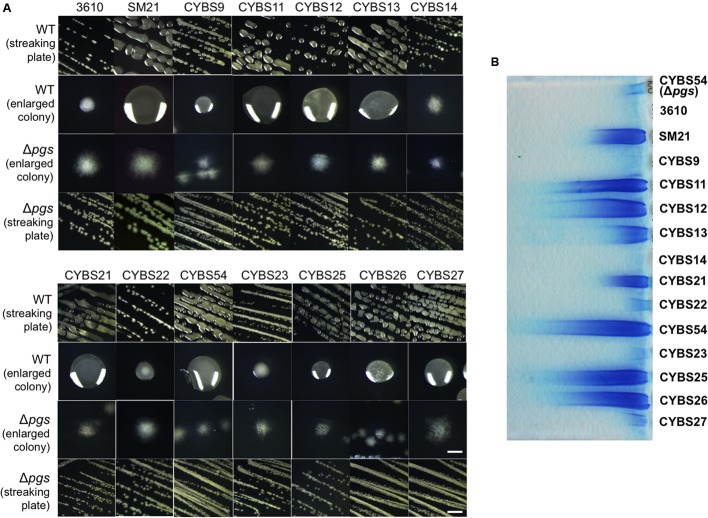
**Different environmental isolates of *B. subtilis* vary significantly in γ-PGA production. (A)** 13 different environmental isolates of *B. subtilis*, together with the model strain 3610, were streaked out on LB agar plates to compare the colony mucoidy phenotype, a qualitative measurement of γ-PGA production. The corresponding Δ*pgs* mutants deficient in γ-PGA production were also streaked out on the same plates. Pictures were taken after 12 h of incubation at 37°C. Both the streaking plates and enlarged colonies were shown. Scale bars in the panels showing the streaking plate and the enlarged colonies represent 5 and 1 mm in length, respectively. This applies to all similar panels in this figure. **(B)** SDS-PAGE for methylene blue stained samples containing purified γ-PGA from the environmental isolates and 3610 (CYBS54 with a deletion mutation in *pgs* was used as a negative control).

In our previous studies, we showed that environmental isolates of *B. subtilis* were capable of robust biofilm formation and that there was a correlation between biofilm robustness and the biocontrol efficacy of those isolates (Chen et al., 2012, 2013). As the starting point of this work, we wanted to test whether those environmental isolates produce γ-PGA, and if so, how γ-PGA production influences biofilm formation. A total of 13 different *B. subtilis* environmental isolates (**Table 1**), together with the model strain 3610 (Branda et al., 2001), were chosen due to their higher than average biocontrol efficacy shown in previous studies (Chen et al., 2012, 2013). The *B. subtilis* 3610 is a commonly used, undomesticated model strain for biofilm studies (Branda et al., 2001). γ-PGA-producing *B. subtilis* strains are known to form mucoid colonies on LB agar plates (Stanley and Lazazzera, 2005). In multiple previous studies, colony mucoidy was used as a qualitative measurement for γ-PGA production (Stanley and Lazazzera, 2005; Cairns et al., 2013; Chan et al., 2014). We therefore streaked out cells on LB plates and visually compared colony mucoidy after 12 h of incubation at 37°C. As shown in **Figure 2A**, large variations in colony mucoidy were observed among the tested strains; colonies of some environmental isolates (e.g., SM21, CYBS11, CYBS12, CYBS21, and CYBS54) appeared to be much more mucoid and bigger in size than others (e.g., CYBS14, CYBS22, and CYBS23). Interestingly, the strain 3610 was much less mucoid compared to other environmental isolates (**Figure 2A**). This result indicated that γ-PGA production varies significantly in different environmental isolates of *B. subtilis.* To confirm that colony mucoidy was indeed due to γ-PGA production, we introduced a deletion mutation in the *pgs* operon, which is responsible for the biosynthesis of γ-PGA in *B. subtilis*, into those isolates. Our results showed that virtually all the deletion mutants had a dry and near flat colony phenotype on LB plates (**Figure 2A**), confirming that the colony mucoidy was solely due to γ-PGA production. Colonies of strong γ-PGA producers (e.g., CYBS54) often expanded further on the agar plates over time, explaining why those colonies were larger in size (**Figure 2A**). In extreme cases, colonies became semi-fluidic and eventually covered the entire plate (data not shown), which implies that under certain conditions, secreted γ-PGA may help the colony expand on a solid surface. This is somewhat similar to the observation in a previous report, in which another secreted polymeric substance, an EPS, was shown to facilitate colony expansion on the solid surface due to the buildup osmotic pressure in the extracellular space of a colony (Seminara et al., 2012).

To further compare γ-PGA production, we followed a published protocol to purify secreted γ-PGA from cells grown in LB broth (Stanley and Lazazzera, 2005). We then ran a SDS-PAGE and visualized those γ-PGA molecules by staining the gel with methylene blue. Our results show that preparations from different environmental isolates contained varied amounts of γ-PGA as judged by the methylene blue staining SDS-PAGE (**Figure 2B**). This observation also largely matched the colony mucoidy phenotype of those isolates (**Figure 2A**). *B. subtilis* is known to produce other types of secreted polymers, such as EPSs and TasA amyloid fibers (Kearns et al., 2005; Branda et al., 2006). To test the specificity of this dye-staining-based technique, we also compared the γ-PGA preparations from the two environmental isolates (CYBS26 and CYBS54) and their corresponding double mutants of Δ*epsH* Δ*tasA*. Our results showed only a mild decrease in the methylene dye staining in the double mutants of Δ*epsH* Δ*tasA* when compared to the preparations from the wild type cells (Supplementary Figure S1). This suggests that the methylene dye staining-based qualitative measurement of γ-PGA production is largely valid.

### Varied γ-PGA Production in Different Isolates Is in Part Due to Differential Expression of γ-PGA Biosynthesis Genes

**FIGURE 3:**
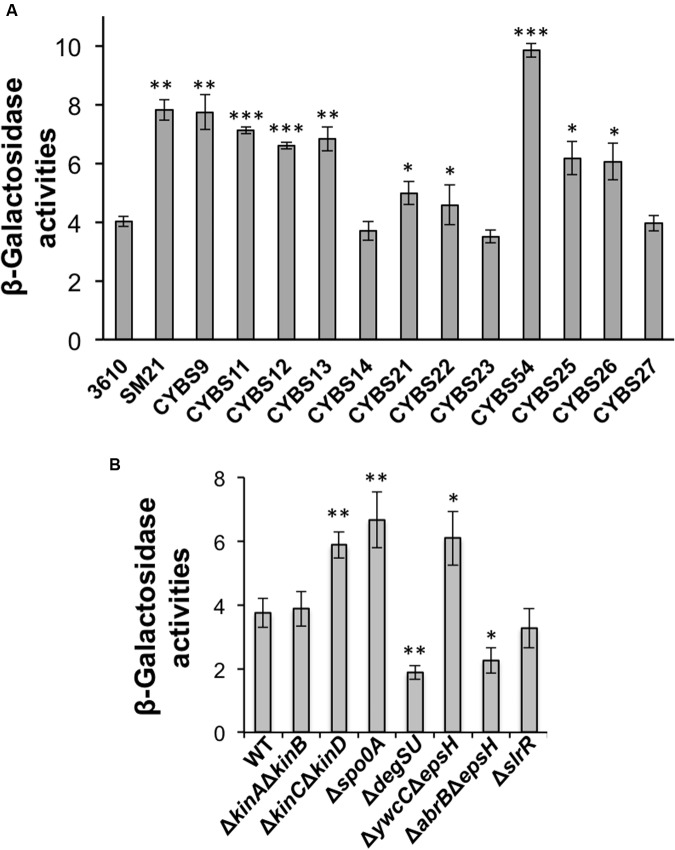
**(A)** Differential expression of the *pgs* genes in different environmental isolates of *B. subtilis*. Assays of β-galactosidase activities from cells of the environmental isolates containing the promoter-*lacZ* transcriptional reporter for the *pgs* operon (P*_pgsB_*-*lacZ*) at the *amyE* locus. Cells were grown in shaking conditions in LB to OD_600_ = 1.0 before harvest. **(B)** Spo0A, YwcC, and DegU regulate the *pgs* genes. Assays of β-galactosidase activities from various mutants in 3610 containing the promoter-*lacZ* transcriptional reporter for the *pgs* operon (P*_pgsB_*-*lacZ*) at the *amyE* locus. Cells were grown in shaking conditions in LB to OD_600_ = 1.0 before harvest. Error bars represent standard deviations of three independent assays. Statistics were analyzed using *t*-test in the R program (https://www.r-project.org/about.html). For values of distinctness (compared to the wild type), one asterisk (^∗^) indicates a *P*-value less than 0.05, two asterisks (^∗∗^) indicates a *P*-value less than 0.01, and three asterisks (^∗∗∗^) indicates a *P*-value less than 0.001.

Although, varied γ-PGA production in different environmental isolates could be due to many different factors, one simple possibility we can think of is the variation in the expression of the *pgs* operon (e.g., due to altered regulation) in different isolates. To test this hypothesis, we applied a promoter *lacZ* fusion for the *pgs* operon [P*_pgsB_*-*lacZ*; note that the promoter sequence of *pgs* was amplified by using 3610 genomic DNA as the template (Gao et al., 2015)], and introduced the reporter fusion into those environmental isolates by genetic transformation. We then compared the activities of the reporter fusion in those isolates grown to late exponential phase (OD_600_ = 1) under shaking conditions. Activities of the P*_pgsB_-lacZ* reporter fusion varied in the tested strains. Interestingly, they largely matched their colony mucoidy phenotype with only a few exceptions (**Figure 3A**). For example, the strain CYBS21 showed much more colony mucoidy than 3610 (**Figure 2A**), yet the activity of the reporter fusion was only marginally higher in CYBS21 than in 3610 (**Figure 3A**).

To further explore the cause for the differential production of γ-PGA in different isolates, we sequenced the *pgs* promoters from five selected environmental isolates of strong (CYBS54 and SM21), intermediate (CYBS9 and CYBS26), or weak (CYBS14) γ-PGA producers based on colony mucoidy (**Figure 2A**). The sequencing results revealed that in the core regions of the *pgs* promoters (∼150-bp upstream of the transcription start covering the -10 and -35 motifs, and the putative DegU binding site in the promoters) (Ohsawa et al., 2009), no variations in DNA sequence was seen among those different strains (Supplementary Figure S2). We did observe some sequence variations further upstream and next to an experimentally characterized CcpA binding site (Ishii et al., 2013). Nevertheless, it will not be straightforward to evaluate the potential significance of these variations in differential γ-PGA production since there are cases that same variations appear in both weak and strong γ-PGA producers (Supplementary Figure S2). As one more interesting observation, the weak γ-PGA producing strain CYBS14 contains a few nucleotide variations in the 5′-untranslated leader sequence of the *pgs* mRNA, which could impact ribosome binding or folding of the mRNA secondary structure. However, and again, this cannot explain why 3610 is also a weak γ-PGA producer but shows no sequence variation in the mRNA leader sequence (Supplementary Figure S2).

To summarize, it is still unclear why γ-PGA production varies significantly in different environmental isolates of *B. subtilis.* It may be in part due to differential expression of the γ-PGA biosynthesis genes as shown in **Figure 3A** (even though all the different strains bear the same promoter fusion), since this result largely matches the colony mucoidy phenotype. This may in turn be due to different activities of the regulatory proteins involved in the *pgs* operon regulation, or due to sequence variations in the promoter regions, which somehow impact transcriptional activation of the *pgs* operon by those proteins. Alternatively, γ-PGA production also depends on the activity of a depolymerase PdgS, whose gene is regulated separately from that of the *pgs* operon (**Figure 1B**; Suzuki and Tahara, 2003).

### KinC and KinD Negatively Regulate γ-PGA Production through Spo0A and AbrB

**FIGURE 4:**
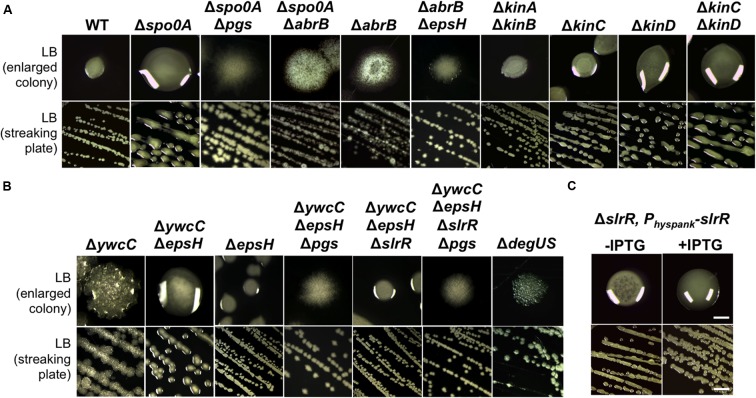
**KinC/KinD and YwcC-mediated pathways regulate γ-PGA production in *B. subtilis*.(A)** Shown is the colony mucoidy phenotype on LB agar media by various mutants in 3610. Strains used in this assay include 3610 (WT), RL4573 (Δ*kinA*Δ*kinB*), RL4262 (Δ*kinC*), RL4569 (Δ*kinD*), RL5273 (Δ*kinC*Δ*kinD*), YY214 (Δ*spo0A*), YY233 (Δ*spo0A* Δ*epsH*), YY208 (Δ*spo0A* Δ*abrB*), YC668 (Δ*abrB*), and YY201 (Δ*abrB* Δ*epsH*). Cells were streaked out on LB plates and incubated for 12 h at 37°C before pictures were taken. Both the streaking plates and enlarged colonies were shown. **(B)** Shown in **(B)** are the same as in **(A)** except that different mutants of 3610 were applied. Strains used here include 3610 (WT), YC295 (Δ*ywcC*), YC299 (Δ*ywcC* Δ*epsH*), RL3852 (Δ*epsH*), YY198 (Δ*ywcC*Δ*epsH*Δ*pgs*), YY199 (Δ*ywcC*Δ*epsH*Δ*slrR*), and YY200 (Δ*ywcC*Δ*epsH*Δ*slrR* Δ*pgs*), and RL4637 (Δ*degSU*). **(C)** The colony mucoidy phenotype by a Δ*slrR* derivative (FC100) expressing *slrR* from an IPTG-inducible *hyperspank* promoter at the *amyE* locus. Scale bars in the panels showing the streaking plate and the enlarged colonies represent 5 and 1 mm in length, respectively. This applies to similar panels in this figure.

The regulation of γ-PGA production in *B. subtilis* is still not completely understood. Previous studies showed that the *pgs* biosynthesis genes were activated by DegU, which was in turn activated by the ComQXPA quorum-sensing system (**Figure 1B**). The colony of the Δ*degSU* double mutant was completely flat with little mucoidy on the LB plate (**Figure 4B**). Interestingly, when working on the *B. subtilis* model strain 3610, we frequently observed that the *spo0A* mutant had a colony mucoidy phenotype on the LB plate (**Figure 4A**), suggesting that Spo0A may negatively regulate γ-PGA production. This colony mucoidy phenotype was completely reversed in the Δ*spo0A*Δ*pgs* double mutant, confirming that Spo0A regulates the *pgs* genes on colony mucoidy (**Figure 4A**). Spo0A is collectively activated by multiple sensory histidine kinases directly or indirectly (from KinA to KinD, **Figure 1A**), whose activities are linked to environmental or plant signals (López et al., 2009; Chen et al., 2012; Beauregard et al., 2013; Shemesh and Chai, 2013). We thus tested whether any of the histidine kinase mutants had a mucoid colony phenotype. Indeed, both the Δ*kinC* and Δ*kinD* mutants in 3610 formed more mucoid colonies on the plates when compared to 3610, especially the double mutant of Δ*kinC*Δ*kinD* (**Figure 4A**). On the contrary, the Δ*kinA* or Δ*kinB* deletion mutation had little influence on the colony phenotype (**Figure 4A**). Spo0A∼P represses the *abrB* gene (Greene and Spiegelman, 1996). A double mutant of Δ*spo0A*Δ*abrB* also lacked the colony mucoidy phenotype, indicating that AbrB is downstream of Spo0A in this regulation. We further ruled out the possibility of a masked effect from overexpression of AbrB-repressed matrix genes since the Δ*abrB*Δ*epsH* double mutant still showed a flat colony on the LB plate.

To summarize, our results indicate that upon sensing environmental signals, KinC and KinD indirectly and negatively regulate γ-PGA production by first activating Spo0A, which subsequently down-regulates AbrB activities. AbrB is a transition stage regulator and a known transcription repressor (Chumsakul et al., 2011). It will be interesting to learn how AbrB positively regulates γ-PGA production. The KinC/KinD-mediated signaling mechanism was previously shown to activate the biofilm pathway in *B. subtilis*; a double mutant of Δ*kinC*Δ*kinD* is severely defective in biofilm formation (Shemesh and Chai, 2013). Thus both biofilm formation and γ-PGA production are co-regulated by the KinC/KinD-mediated signal transduction mechanism but in an opposite fashion.

### The TetR-Type Transcription Repressor YwcC also Regulates γ-PGA Production

YwcC is a TetR-type transcription repressor (Kobayashi, 2008; Chai et al., 2009). Previous studies showed that in *B. subtilis*, YwcC negatively regulates matrix genes and thus biofilm formation via SlrA and SlrR, two antagonist proteins of the biofilm master repressor SinR (**Figure 1A**; Kobayashi, 2008; Chai et al., 2009, 2010). Interestingly, we found that the Δ*ywcC* mutant in 3610 also had a colony mucoidy phenotype (**Figure 4B**), indicating that like KinC/KinD and Spo0A, YwcC is also involved in co-regulation of both matrix genes and genes for γ-PGA biosynthesis. Again, the colony mucoidy of the Δ*ywcC* mutant was not likely due to overexpression of YwcC-controlled matrix genes, since the colony of the Δ*ywcC*Δ*epsH* double mutant was still very much mucoid when compared to the Δ*epsH* mutant (**Figure 4B**). On the other hand, colonies of the Δ*ywcC*Δ*epsH*Δ*pgs* triple mutant completely lost colony mucoidy, suggesting that the colony mucoidy phenotype seen in the Δ*ywcC*Δ*epsH* double mutant was due to over-production of γ-PGA (**Figure 4B**).

YwcC negatively regulates the *slrR* gene, which encodes a key regulatory protein controlling matrix production (Kobayashi, 2008; Chai et al., 2009). To test whether SlrR is involved in γ-PGA production controlled by YwcC, we introduced a Δ*slrR* deletion mutation into the double mutant of Δ*ywcC*Δ*epsH*. The triple mutant formed colonies that were less mucoid than that of Δ*ywcC*Δ*epsH* (**Figure 4C**), indicating that SlrR may lie downstream of YwcC in regulating γ-PGA production and colony mucoidy. To further confirm that SlrR (positively) regulates γ-PGA production, we applied a Δ*slrR* derivative with an inducible *slrR* gene (under the control of the *hyperspank* promoter) at an ectopic locus (Chai et al., 2010). When the inducer IPTG was added to the LB agar media, the resulting colonies were more mucoid than the ones in the absence of IPTG (**Figure 4C**). This confirms that SlrR is a positive regulator for γ-PGA production. To conclude, we believe that YwcC and SlrR mediate another pathway co-regulating both biofilm matrix genes and the γ-PGA biosynthetic genes. The activity of YwcC is predicted to be regulated by binding to a small ligand (a chemical signal) from the environment or plant host (Kobayashi, 2008; Chai et al., 2009). While the signal is unknown, we speculate that it may be important for both biofilm formation and γ-PGA production.

Finally, to test whether the colony mucoidy phenotype seen in various mutants of *B. subtilis* 3610 is caused by altered expression of the γ-PGA biosynthetic genes in those mutants, we introduced a promoter-*lacZ* transcriptional fusion for the *pgs* operon (P*_pgsB_*-*lacZ*) into various mutants and performed assays of the β-galactosidase activities for the resulting strains (Gao et al., 2015). As shown in **Figure 3B**, the Δ*kinC*Δ*kinD* double mutant, the Δ*spo0A* mutant, and the Δ*ywcC* mutant all had higher activities of the transcriptional fusion whereas the Δ*degUS* mutant and the Δ*abrB*Δ*epsH* mutant showed a reduced activity of the reporter (**Figure 3B**; Ohsawa et al., 2009; Chan et al., 2014; Marlow et al., 2014). The Δ*slrR* mutant did not show a clearly reduced activity in the reporter fusion (**Figure 3B**). However, it is known from previous studies that in LB, the *slrR* gene is expressed weakly and only in a small proportion of cells (Chai et al., 2010). Indeed, our unpublished RNA-seq results suggest in SlrR-overproducing (P*_hyspank_*-*slrR*) cells, the *pgs* operon is among the highly induced genes (data not shown). Overall, the colony mucoidy phenotype in different mutants of 3610 matched well the levels of the expression of the *pgs* operon in those strains.

### γ-PGA Contributes to Complex Morphology and Robustness of the Colony Biofilms in Different Environmental Isolates of *B. subtilis*

**FIGURE 5:**
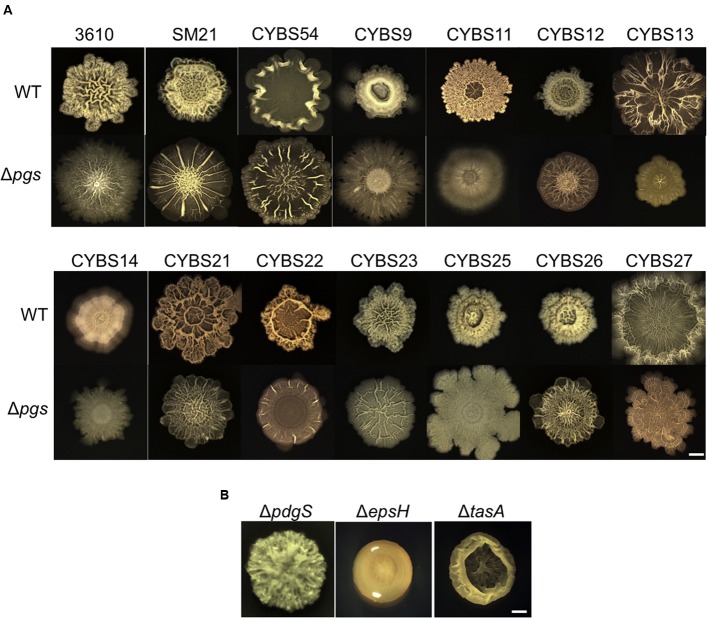
**Poly-γ-glutamic acid production influences robustness and complex morphology of the colony biofilms in the environmental isolates of *B. subtilis*. (A)** Colony biofilms formed by various environmental isolates of *B. subtilis* and the corresponding Δ*pgs* mutants. Colony biofilms were grown on LBGM for 48 h at 30°C before pictures were taken. Scale bar: 5 mm. **(B)** Colony biofilms formed by three 3610 derivatives, YY54 (Δ*pgdS*), RL3852 (Δ*epsH*), and SB505 (Δ*tasA*). Scale bar: 5 mm.

Given that γ-PGA is produced in abundance by many environmental isolates of *B. subtilis* (**Figure 2A**) and that the same isolates are capable of forming robust biofilms (Chen et al., 2013), it is reasonable for us to ask whether γ-PGA production contributes to biofilm formation in those strains. Although, several previous studies already investigated the possible role of γ-PGA in biofilm formation in *B. subtilis* (Stanley and Lazazzera, 2005; Branda et al., 2006; Morikawa et al., 2006), due to different strains, medium conditions, and experimental settings used in those studies, the conclusions were inconsistent. Therefore, it remains unclear whether γ-PGA is part of the biofilm matrix and whether its production is important for biofilm formation. Here, we tested the colony biofilm phenotype for the 13 wild strains and the corresponding Δ*pgs* mutants in the biofilm-inducing medium (LBGM) (Shemesh and Chai, 2013). Most environmental isolates as well as *B. subtilis* 3610 formed colony biofilms with distinct surface architectures (**Figure 5A**). Many of the corresponding Δ*pgs* mutants, however, formed colony biofilms with less structural complexity (e.g., CYBS11, CYBS13, CYBS22, and CYBS25). In the well-studied *B. subtilis* model strain 3610, lack of complex surface features is often used as an indication of weaker or defective biofilms. For example, the colonies biofilms formed by the *eps* or *tasA* mutants that are deficient in biofilm matrix production are also featureless (**Figure 5B**). Our results thus suggest that in general, γ-PGA production plays a notable role in increasing complex morphology and robustness of colony biofilms in many of the tested environmental isolates, although less important than the EPSs and TasA fibers. As one more piece of evidence, the colony biofilm by the *pdgS* mutant of *B. subtilis* 3610 (which is deficient in production of the γ-PGA depolymerase and shown to have a higher yield of γ-PGA) demonstrated an increased structural complexity (**Figure 5B**), similar to what was seen in the Δ*sinR* mutant (Subramaniam et al., 2013). In terms of the putative role of γ-PGA in biofilm formation, since there is no evidence that γ-PGA can influence expression of the matrix genes such as the *epsA-O* and *tapA* operons (data not shown), we speculate that secreted γ-PGA may become part of the biofilm matrix or facilitate assembly of other matrix components. In a previous study (Branda et al., 2006), it was shown that the TasA fibers and the EPS can complement extracellularly in that a mixture of the Δ*tasA* and Δ*epsH* mutant cells are capable of forming wild type-like biofilms. We did similar complementation experiments by mixing the *pgs* mutant cells with an equal number of either Δ*tasA* or Δ*epsH* cells (Supplementary Figure S3). Our results seem to indicate that like TasA and EPS, γ-PGA can also be shared by cells within the biofilm.

### Influence of γ-PGA Production on *B. subtilis* Biofilm Formation Can be Medium-Dependent

**FIGURE 6:**
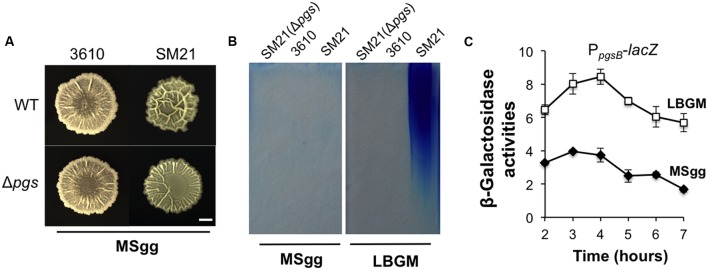
**The influence of γ-PGA production on colony biofilm robustness is medium-dependent. (A)** The colony biofilm phenotype on MSgg from the wild type strains and the *pgs* mutants in both 3610 and SM21. Scale bar: 5 mm in length. **(B)** SDS-PAGE for methylene blue stained samples containing purified γ-PGA from YY170 (Δ*pgs* in SM21, used as a negative control), 3610, and SM21, prepared from cells growing in either LBGM or MSgg. **(C)** Assays of β-galactosidase activities from a SM21 derivative (YY245) containing the promoter-*lacZ* transcriptional reporter for the *pgs* operon (P*_SM21pgsB_*-*lacZ*) at the *amyE* locus show different expression of the reporter in LBGM and MSgg. Cells were grown in shaking conditions in LBGM (unfilled squares) or MSgg (filled diamonds) broth over a period of 7 h. Assays were done in triplicate. Error bars represent standard deviations.

A puzzle thus arose since in a previous study (Branda et al., 2006), it was reported that the Δ*pgs* mutant of *B. subtilis* 3610 showed no difference in the biofilm phenotype from that of the wild type strain. However, it is important to note that biofilm assays in that study were conducted in MSgg, another commonly used biofilm-inducing medium for *B. subtilis* (Branda et al., 2001). We repeated the assay for *B. subtilis* 3610 and the corresponding Δ*pgs* mutant in MSgg. Not surprisingly, little difference in colony biofilm phenotype was observed between the two strains in MSgg (**Figure 6A**). We also repeated this test by using SM21, a stronger γ-PGA producer (**Figure 2A**) and its corresponding Δ*pgs* mutant. The result was similar although this time a mild difference was seen between the two strains (**Figure 6A**). We already showed that in LBGM, the difference in colony morphology and robustness was quite clear between these two wild type strains and the corresponding Δ*pgs* mutants (**Figure 5A**). It seems to us that the role of γ-PGA in biofilm formation in tested *B. subtilis* strains somehow depends on what media we use for the biofilm assays.

Although, the above results may address much of the discrepancy in previous results about the role of γ-PGA in biofilm formation, the cause of the observed medium-dependence is still unknown. One possibility could be that in MSgg, the expression of the *pgs* operon is significantly lower than in LBGM and thus the importance of γ-PGA in *B. subtilis* biofilm formation diminishes in MSgg. To test that, we performed two assays, comparing both the *pgs* gene expression and γ-PGA production in LBGM and MSgg using two selected strains (SM21 and 3610). In the first experiment, we prepared secreted γ-PGA from cells grown in either LBGM or MSgg and examined the samples on SDS-PAGE similarly as described above. Our results confirmed that cells of the strong γ-PGA producer SM21 produced much higher amounts of γ-PGA in LBGM than in MSgg, while the weaker γ-PGA producer 3610 did not seem to have a strong production of γ-PGA in either medium (**Figure 6B**). In the second experiment, we tested whether the *pgs* genes were differentially expressed in LBGM and MSgg. A promoter-*lacZ* reporter fusion (P*_SM21pgsB_*-*lacZ*) was similarly constructed and introduced into SM21 as described in a previous study (Gao et al., 2015) except that this time the promoter sequence was PCR amplified using SM21 genomic DNA as the template. As shown in **Figure 6C**, in SM21, the *pgs* reporter fusion were expressed significantly higher in LBGM (unfilled squares) than in MSgg (filled diamonds). A similar difference in *B. subtilis* 3610 between the two different media was previously reported by us (Gao et al., 2015). In conclusion, we believe that γ-PGA production plays a notable role in biofilm formation in *B. subtilis* and that more importantly conditions strongly influencing the yield of γ-PGA production may dictate the importance of γ-PGA in *B. subtilis* biofilm formation.

### Bacterial Root Colonization Efficiency Positively Correlates with γ-PGA Production in Tested *B. subtilis* Strains

**FIGURE 7:**
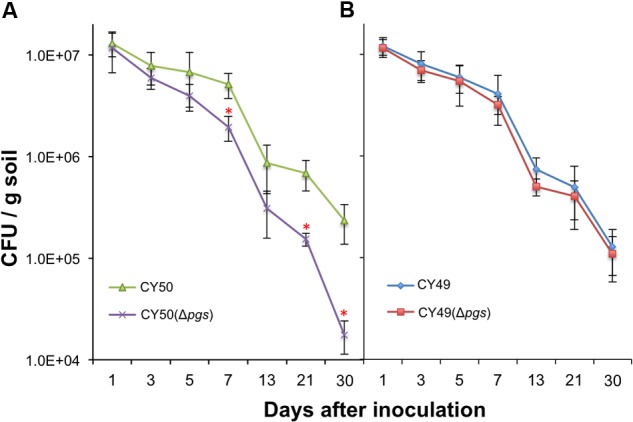
**Poly-γ-glutamic acid plays a notable role in plant root colonization in selected environmental isolate.** Assays of tomato plant root colonization by a CYBS54 derivative (CY50, **A**), the *pgs* mutant in the CYBS54 derivative (CY151, **A**), and a 3610 derivative (CY49, **B**), the *pgs* mutant in the 3610 derivative (CY150, **B**) over a period of 30 days under greenhouse conditions. Root colonization efficiency was counted as C.F.U of root-associated *B. subtilis* cells per gram of collected soil. Error bars represent standard deviations from four independent experiments. Statistics were analyzed using *t*-test in the R program (https://www.r-project.org/about.html). For values of distinctness (compared to the wild type), the asterisk (^∗^) indicates a *P*-value less than 0.05.

The physiological function of γ-PGA in *B. subtilis* has been investigated in previous studies (Ogunleye et al., 2015) but remains unclear. Since *B. subtilis* is a soil bacterium and can secrete γ-PGA in large quantities, we suspect that one of the important functions of γ-PGA may have to do with environmental fitness of the bacterium or interactions with the plant, the natural host of *B. subtilis* in the rhizosphere. This is analogous to the role of γ-PGA in *B. anthracis* in bacteria–host interactions and virulence (Jang et al., 2011). We further postulate that γ-PGA may directly contribute to colonization of plant roots by the bacteria or do so indirectly through increasing biofilm robustness [In a previous study, we demonstrated that biofilm formation plays an essential role in root colonization by *B. subtilis* cells (Chen et al., 2013)]. To test our hypothesis, we performed colonization assays on the tomato plant root using the wild type strains and the Δ*pgs* mutants from both a 3610 derivative (a weak γ-PGA producer, **Figure 2A**) and a CYBS54 derivative (a strong γ-PGA producer, **Figure 2A**). The assays were done over a period of 30 days under green house conditions by following a protocol that we established in the previous study (Chen et al., 2013). On various days following bacterial inoculation, root-associated bacterial cells were isolated and total C.F.U was counted by plating. Our results showed that for the CYBS54 derivative (a stronger γ-PGA producer), the wild type cells had a 12-fold higher attachment efficiency than the Δ*pgs* mutant at the end of the experiment (**Figure 7A**). For the 3610 derivative, the number of the wild type cells attached to the roots was only marginally higher than that of the Δ*pgs* mutant (**Figure 7B**). This indicates that for strong γ-PGA-producing strains, γ-PGA may play an important role in bacterial attachment to the plant root surface.

## Discussion

Various soil-born *Bacillus* species are capable of producing γ-PGA (Gardner and Troy, 1979; Makino et al., 1989; Stanley and Lazazzera, 2005; Ogunleye et al., 2015). One of the well-studied examples is *B. anthracis*, which produces γ-PGA as a cell-surface anchored polymer and a component of the capsule. The γ-PGA capsule protects *B. anthracis* cells from phagocytosis, disguises the bacterium from immune surveillance, and enhances *B. anthracis* toxin activities during host infections (Jang et al., 2011). The function of γ-PGA has also been investigated in *B. subtilis*, however the physiological role of γ-PGA remains largely unclear. For one example, past studies on the putative role of γ-PGA in biofilm formation in *B. subtilis* led to contradictory conclusions due to usage of different strains, media, and biofilm assays in those studies (Stanley and Lazazzera, 2005; Branda et al., 2006; Morikawa et al., 2006). In this study, we showed that strong γ-PGA production contributed to the complex morphology and robustness of the colony biofilms in many of the tested environmental isolates of *B. subtilis*. This is supported by the observation that the biofilm phenotypes between the wild type strains and the Δ*pgs* mutants are different. We also verified that different media conditions (e.g., LBGM and MSgg) could materially influence the expression of the γ-PGA biosynthesis genes and therefore γ-PGA production, so that under some growth conditions (such as in MSgg), the difference in the biofilm phenotype between the wild type and the Δ*pgs* mutant tended to be mild or minimal. Our results and interpretation largely addressed the discrepancy in the results obtained from several previous studies. In terms of why the *pgs* genes are differentially expressed in different media, one recent report showed that presence of glutamic acids repressed the expression of the *pgs* genes in both *B. subtilis* and *Bacillus thuringiensis* (Kambourova et al., 2001). Interestingly, MSgg contains a large amount of glutamic acid (0.5%, v/v), which for still unknown reasons is essential for the biofilm-inducing activity of the medium (Branda et al., 2001). In this work, we also presented evidence that γ-PGA plays an important role in the process of root colonization by the bacterium. Since, γ-PGA is a secreted polymer in large quantities, we argue that one of the primary functions of γ-PGA may have to do with bacteria–plant interactions. Various *B. subtilis* strains have been used as the BCAs for plant protection. Our unpublished data also suggest γ-PGA may play a role in plant protection against certain pathogenic species since the Δ*pgs* mutants were less effective in plant protection (data unpublished, Y. Chai). However, this effect could be indirectly due to the role of γ-PGA in biofilm formation and root colonization. In the future, it will be interesting to carry out more detailed studies on how γ-PGA contributes to *B. subtilis*–plant interactions.

Many bacteria produce different kinds of secreted polymeric substances for different purposes. Since production of such polymers is often energy costly, it is important for bacterial cells to coordinate the production of these polymers at various levels. Here, we found that genes for production of γ-PGA and those for biofilm matrix seem to be inversely regulated by at least two independent pathways. One pathway involves sensory histidine kinases KinC/KinD and transcription regulators Spo0A and AbrB. This pathway positively regulates biofilm formation while simultaneously and negatively controls γ-PGA production, as revealed in the study (**Figure 1C**). Thus, our results imply an interesting switch-like mechanism for the control of both matrix production and production of γ-PGA, trigged by environmental or plant signals sensed by KinC and KinD. Previous studies have shown that KinC and KinD were activated by plant-derived polysaccharides and malic acids (Chen et al., 2012; Beauregard et al., 2013). The second pathway for the inverse regulation is mediated by the previously described DegS-DegU two-component system, which positively regulates γ-PGA production but strongly represses the biofilm matrix genes (**Figures 1B,C**; Marlow et al., 2014). Whether and how DegS-DegU is regulated by environmental or host signals is not known, but highly likely since the sensory kinase DegS contains an extracellular sensing domain for putative environmental signals (Jers et al., 2011).

To add another layer of complexity to the regulation of γ-PGA production, two recent studies independently showed that a deletion mutation in the *motA* gene encoding the flagella motor protein resulted in a strong colony mucoidy phenotype (Cairns et al., 2013; Chan et al., 2014). Our own independent investigation prior to those publications also led to similar observations (Supplementary Figure S4). The authors in the published studies further showed that the phenotype was likely due to altered flagella motion, not necessarily the flagella apparatus since mutations in the genes such as *hag*, which encodes the flagellin protein, did not cause a similar colony mucoidy phenotype (Cairns et al., 2013; Chan et al., 2014). In the Δ*motA* mutant, the DegS-DegU two-component system was highly activated, which led to higher expression of the γ-PGA biosynthesis genes. This may well-explain the colony mucoidy phenotype in the Δ*motA* mutant. What remains unclear is how block of the flagella motion activates the DegS-DegU two-component system. This implies that the γ-PGA production in *B. subtilis* is also a response to biophysical or mechanical signals generated through cells interacting with the environmental space. The interaction between secreted polymers and flagella motion has also been investigated in other bacterial systems in recent studies. For example, in *Salmonella*, it has been shown that the accumulation of the cellulose polymers around the cells hindered flagella rotation without affecting gene expression or assembly of the flagella (Zorraquino et al., 2013). This functions as a control mechanism for motility in response to cyclic-di-GMP mediated cytoplasmic sensing pathway. However, here we did not find evidence that γ-PGA production is able to impact flagella-dependent swarming motility on the semi-solid surface (unpublished data).

Finally, the regulation by YwcC-mediated pathway on both matrix genes and γ-PGA biosynthesis genes is interesting because this pathway activates both groups of genes, different from the opposing regulation mediated by either DegU or Spo0A. In one of our own studies investigating the function of YwcC (Chai et al., 2009), we hypothesized that YwcC controls an emergency pathway for biofilm induction in *B. subtilis*. That is, this pathway is normally silent, however, when YwcC is antagonized by a yet-unknown environmental or host signal, all cells start to strongly express matrix genes, resulting in hyper-robust biofilms in a relatively short time [note that in Spo0A-mediated biofilm activation, only a subpopulation of cells strongly but gradually produce biofilm matrix, which is nevertheless shared by the entire community (Chai et al., 2008)]. We can imagine that under certain environmental conditions, cells will need to strongly produce both γ-PGA and biofilm matrix in order to quickly establish close bacteria–plant interactions. In conclusion, our studies outline an important role of γ-PGA in biofilm formation, bacteria–plant interactions, and possibly environmental survival in *B. subtilis*. We also propose an interesting interplay between the production of γ-PGA and that of biofilm matrix during bacteria–plant interactions in the rhizosphere. This may be another example of how bacteria sequentially utilize different secreted polymeric substances at different stages of bacteria–plant interactions, which have been well-documented in *Rhizobium* species (Long, 1989).

## Author Contributions

YChen, J-HG, and YChai designed the experiments. YY, FY, YChen, and CJ performed the experiments. YY, FY, J-HG, and YChai analyzed the results and wrote the manuscript.

## Conflict of Interest Statement

The authors declare that the research was conducted in the absence of any commercial or financial relationships that could be construed as a potential conflict of interest.
