# Supplementary material for: Poly-γ-Glutamic Acids Contribute to Biofilm Formation and Plant Root Colonization in Selected Environmental Isolates of Bacillus subtilis
Source: Front Microbiol. 2016 Nov 10;7:1811. doi: 10.3389/fmicb.2016.01811 (PMC5102903; doi:10.3389/fmicb.2016.01811)
Supplement: Supplementary file 1 [file Data_Sheet_1.pdf]

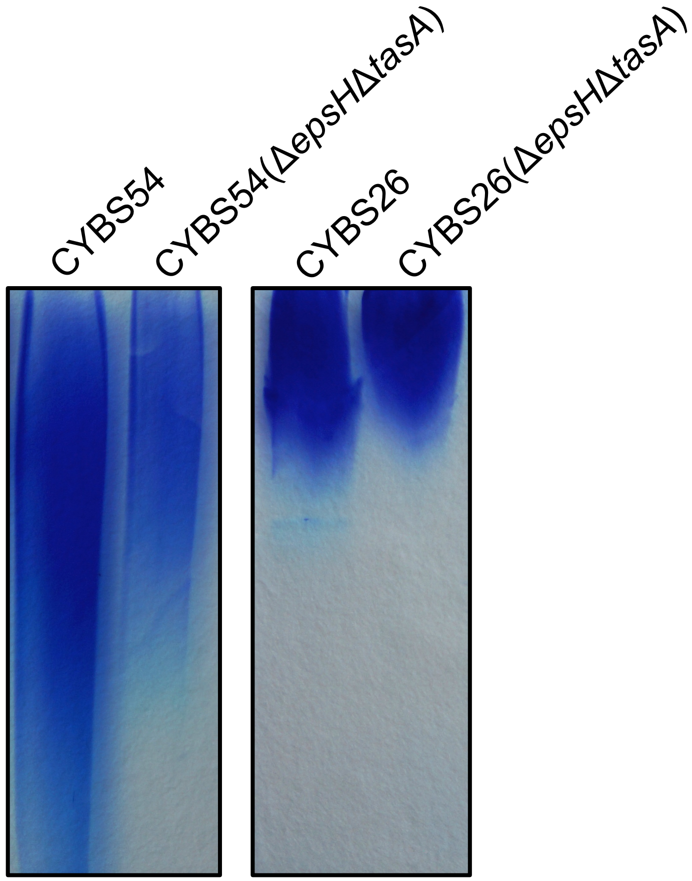

Supplemental Figure 1

```
3610    GATTGATTTGAAATGCATATTAAATACAAACAGCAGGGTGTGACTATACGTCCAGAAGGATATCAGGAGAAAAAT
SM21    GATTGATTTGAAATGCATATTAAACACAAACACCAGGGTGTGACTATACGTCCAGAAGGATATCAGGGGAAAAAT
CYBS9   GATTGATTTGAAATGCATATTAAACACAAACACCAGGGTGTGACTATACGTCCAGAAGGATATCAGGGGAAAAAT
CYBS14  GATTGATTTGAAATGCATATTAAATACAAACAGCAGGGTGTGACTATACGTCCAGAAGGATATCAGGAGAAAAAT
CYBS26  GATTGATTTGAAATGCATATTAAATACAAACAGCAGGGTGTGACTATACGTCCAGAAGGATATCAGGAGAAAAAT
CYBS54  GATTGATTTGAAATGCATATTAAATACAAACAGCAAGGTGTGACTATACGTCCAGAAGGATATCAGGAGAAAAAT
        ************************.*******.**:*******************************.*******

          CcpA
        <-------->
3610    GAAAGCGCATTCTCTTTGATTTTTTTAAAAAGTGATAAGGTATAAGTCCTGCTGTTTTCCAAAAATAGAAAACAG
SM21    GAAAGCGCATTCTCTTCGATTTTTAAAAAAAGTGATGAGGTATAAGTCCTGCTGTTTTCCAAAAATAGAAAACAG
CYBS9   GAAAGCGCATTCTCTTCGATTTTTAAAAAAAGTGATGAGGTATAAGTCCTGCTGTTTTCCAAAAATAGAAAACAG
CYBS14  GAAAGCGCATTCTCTTCGATTTTTAAAAAAAGTGATAAGGTGTAAGTCCTGCTGTTTTCCAAAAATAGAAAACAG
CYBS26  GAAAGCGCATTCTCTTCGATTTTTAAAAAAAGTGATAAGGTGTAAGTCCTGCTGTTTTCCAAAAATAGAAAACAG
CYBS54  GAAAGCGCATTCTCTTTGATTTTTAAAAAAAGTGATAAGGTGTAAGTCCTGCTGTTTTCCAAAAATAGAAAACAG
        ****************.*******::**********.****.*********************************

                                                                            DegU
                                                                         <------
3610    TTTGTAGGTATAAAATCTCTTTCAAAAGAGAAGTTTGGCTTAGTCGATTAGGGAAGATTATGTTACATAATGCCG
SM21    TTTGTAGGTATAAAATCTCTTTCAAAAGAGAAGTTTGGCTTAGTCGATTAGGGAAGATTATGTTACATAATGCCG
CYBS9   TTTGTAGGTATAAAATCTCTTTCAAAAGAGAAGTTTGGCTTAGTCGATTAGGGAAGATTATGTTACATAATGCCG
CYBS14  TTTGTAGGTATAAAATCTCTTTCAAAAGAGAAGTTTGGCTTAGTCGATTAGGGAAGATTATGTTACATAATGCCG
CYBS26  TTTGTAGGTATAAAATCTCTTTCAAAAGAGAAGTTTGGCTTAGTCGATTAGGGAAGATTATGTTACATAATGCCG
CYBS54  TTTGTAGGTATAAAATCTCTTTCAAAAGAGAAGTTTGGCTTAGTCGATTAGGGAAGATTATGTTACATAATGCCG
        ***************************************************************************

        --> -35                  -10          +1
3610    ATTGAGAATTCATAGTGATTCTATATACTGATGAATGAATTTACAACAATATAGAAGGAGATGTCGAAAAGCA
SM21    ATTGAGAATTCATAGTGATTCTATATACTGATGAATGAATTTACAACAATATAGAAGGAGATGTCGAAAAGCA
CYBS9   ATTGAGAATTCATAGTGATTCTATATACTGATGAATGAATTTACAACAATATAGAAGGAGATGTCGAAAAGCA
CYBS14  ATTGAGAATTCATAGTGATTCTATATACTGATGAATGAATTTACACCA-TATAGAAGGAGATGTCGAAAAGCA
CYBS26  ATTGAGAATTCATAGTGATTCTATATACTGATGAATGAATTTACAACAATATAGAAGGAGATGTCGAAAAGCA
CYBS54  ATTGAGAATTCATAGTGATTCTATATACTGATGAATGAATTTACAACAATATAGAAGGAGATGTCGAAAAGCA
        *********************************************.**:************************
```

Supplemental Figure 2

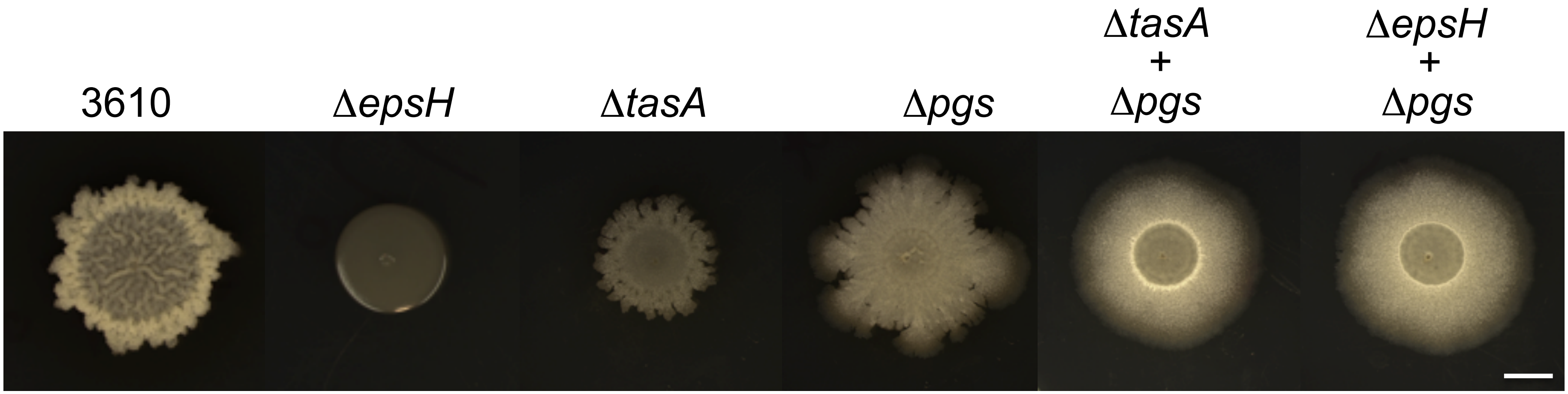

Supplemental Figure 3

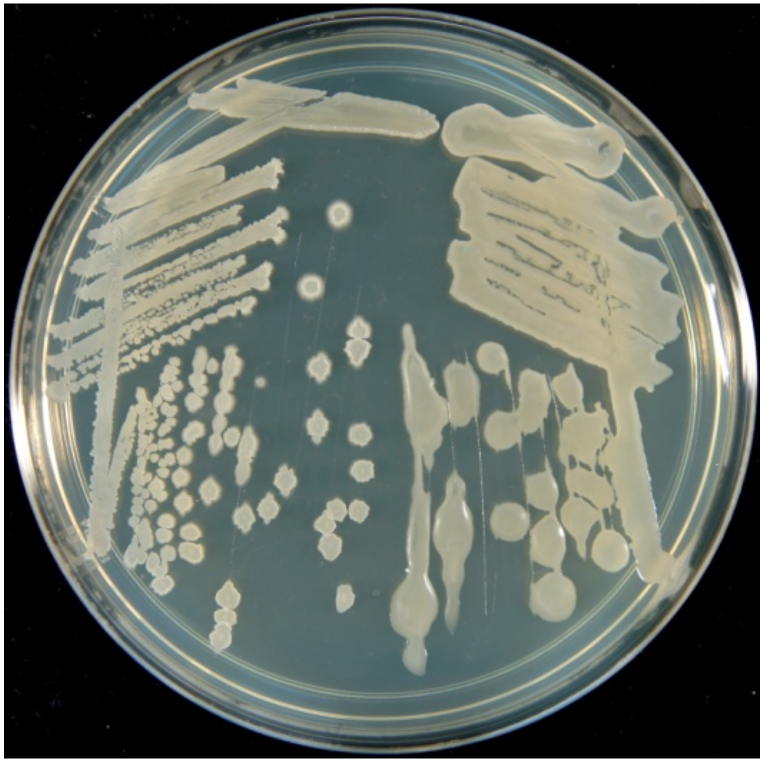

3610 Δ*motA*

Supplemental Figure 4

Supplemental figure legends

Fig. S1. SDS-PAGE for methylene blue stained samples containing purified γ-PGA from the environmental isolates CYBS54, CYBS26 and their corresponding Δ*epsH*Δ*tasA* double mutants. Purification of PGA was described in the Methods.

Fig. S2. DNA sequence alignment of the *pgs* promoter regions from various *B. subtilis* environmental strains. The *pgs* promoter sequences were obtained by PCR amplification of the corresponding regions using genomic DNAs from selected *B. subtilis* environmental strains and primers P_{pgsA}-F1 and P_{pgsA}-R1 (Table S1) and by DNA sequencing. The DNA sequence alignment was performed by using the program ClustalW (http://www.genome.jp/tools/clustalw/). The columns with nucleotide polymorphism are highlighted in yellow and green. Yellow represents consensus while green indicates changes. The -10 and -35 motifs of the sigma A-dependent promoter, and the transcription start (+1) were experimentally confirmed and are indicated here (1). A CcpA binding site (*cre* box) was also experimentally characterized in a previous study (2)and indicated here.

Fig. S3. Biofilm complementation by various matrix mutants. Shown here are colony biofilms on LBGM agar plates formed by the wild type(3610), the Δ*epsH* mutant (RL3852), the Δ*tasA* mutant(SB505), the Δ*pgs* mutant(FY6), a 1:1 mixture of the Δ*tasA* and Δ*pgs* mutants, and a 1:1 mixture of Δ*epsH* and Δ*pgs* mutants. 2-ul log phase cells were spotted onto the center of the plates and incubated at 37°C for 2 days before images were taken. The scale bar represents 5 mm in length.

Fig. S4. The Δ*motA* mutant shows a colony mucoidy phenotype. The wild type strain (3610) and the Δ*motA* mutant in 3610 (CY258) were streaked out on the LB agar plate and incubated for 16 hours at 37°C before the picture was taken.

Supplemental references

1. **Ohsawa T, Tsukahara K, Ogura M.** 2009. *Bacillus subtilis* Response Regulator DegU Is a Direct Activator of *pgsB* Transcription Involved in γ-Poly-glutamic Acid Synthesis. Bioscience, Biotechnology, and Biochemistry **73:**2096-2102.

2. **Ishii H, Tanaka T, Ogura M.** 2013. The *Bacillus subtilis* Response Regulator Gene degU Is Positively Regulated by CcpA and by Catabolite-Repressed Synthesis of ClpC. Journal of Bacteriology **195:**193-201.

Table S1. Oligonucleotides used in this study.

Primer name	Primer sequence (5'-3')
pdgS-P1	AAGATACGCTTGGAGAATTTGCGAAGCAAA
pdgS-P2	CAATTCGCCCTATAGTGAGTCGTTTTTATTATCTCCTCCTC
pdgS-P3	CCAGCTTTTGTTCCCTTTAGTGAGAAGTATACGGGTGCAATA
pdgS-P4	CGATTTAGGTGTATAATGAACGGGATGGCG
pgs-P1	CGCCGTTAAATCGGTCTTGAGCG
pgs-P2	CAATTCGCCCTATAGTGAGTCGTGTATGACAGCACAGGCTA
pgs-P3	CCAGCTTTTGTTCCCTTTAGTGAGAAGTATACGGGTGCAATA
pgs-P4	CGATTTAGGTGTATAATGAACGGGATGGCG
PpgsB-F1	GTACAAGCTTATGACCTTGTCCTTAAGAAACAG
PpgsB-R1	GTACGGATCCCTTCTATATTGTTGTAAATTC
